# Salvage of fractured abutment screw by transfer cap screw replacement (original study)

**DOI:** 10.1002/ccr3.2159

**Published:** 2019-05-10

**Authors:** Rawaa Y. Al‐Rawee, Fatima A. Mohammad, Bashar A. Tawfeeq

**Affiliations:** ^1^ Department of Oral and Maxillofacial Surgery Al‐Salam Teaching Hospital Mosul Iraq

**Keywords:** abutment screw fracture, biomechanics effect, dental implant complication, overload forces, prosthetic complications

## Abstract

Salvage of fractured abutment screw is one of the critical complications, which can be solved in different ways. In this study, an innovative approach is introduced to solve this problem. It is a relatively simple, non‐invasive technique, which would provide an excellent result when executed with ultimate care and precision.

## 1 INTRODUCTION

In implantology, much clinical research has focused on the associations between fixtures and occlusal loading, during the past three decades in implant dentistry.^1^

Extensive occlusal stress with peri‐implantitis is the major cause of implant failure. Implantologist knew the late failures arise from pathological processes involving biomechanics and host‐parasite interactions.^2^ Abutment screw fracture is one of the causes of late prosthetic failures. Biomechanical problems involve the interface between the individual fixture, the abutment, and the prosthesis.^3^ Clinical observations referred to the screw fractures have lately garnered increasing attention.^4^

Abutment screw fracture is a rare event, occurring <0.5%. Nonetheless, when a fracture occurs, it is very disturbing for the clinician.^5^ Prosthodontically, functional cyclic loading can end up with a fractured screw. Many researches prove that perspective point. When the abutment screw was overloaded and fractured, leaving the abutment and coronal screw fragment outside the fixture, the apical fragment will be stuck in the fixture itself.

Every dentist knew that each fixture has a crest module, body, and apical region. The crest module of a fixture body is the portion designed to retain the prosthetic component. An antirotation feature is included in the crest module.^6^, ^7^

Prosthetic screw: It is a fixture component that secures the fixture‐abutment or prosthesis to the fixture body. Loosening of the screw may result in the displacement of the prosthesis and eventual loss of prosthetic function.

Torque is defined as the movement of a system of forces producing rotation.^8^ The torque applied to an abutment screw has a clamping effect, called the preload. It holds the abutment to the fixture.^9^

Occlusal loading is a multidirectional and variable magnitude force. There is a slight wobble of the abutment during functional loads.^10^, ^11^ Fatigue fracture can be induced by the tensile and bending movements, which are applied on the fractured abutment screw. This event will end up with the dislodgment of the crown leaving the fractured piece in the fixture.^12^

Fatigue fracture is caused by biomechanical overload, improper placement techniques, and a nonpassive fit of the suprastructure errors. Extended period of clinical use, repeated retightening of loose screw, poor design of the fixture‐abutment connection, inadequate tightening and screw joint movement, and metal fatigue after screw loosening also can induce these complications.^13^, ^14^, ^15^, ^16^

Screw fracture is a challenge for the clinician due to the difficulty of removal of fractured screw fragments. A review of in vivo butt‐joint fixture studies about abutment screw loosening stated that screw loosens when the joint‐separating force is high. Forces acting to separate the screw joint are greater than the forces keeping the abutment and fixture together (clamping force).^17^ Off‐axial occlusal loading to the fixture is an example of forces acting to weaken the abutment‐fixture screw joint.

Additionally, the surface of a new metal screw has microscopic imperfections in the form of high spots, grooves, and irregularities, such that, when initial torque is applied, only the high spots in the system will be in contact. Flattening and wear of these high spots are described as screw settling and will result in the loss of the initial preload.^18^ When a screw loosens, surface damage occurs at high‐stress locations, particularly at the screw head and the first thread locations.

There are several techniques for managing a fractured abutment screw.^19^, ^20^ These include the following:

- Screw fragment retrieval, replaced by another screw.
- Fixture removal or discard and reimplantation in another site if applicable.
- Fabrication of a cemented cast post and core.

In lucky circumstances, the stock fractured end can be retrieved and replaced by a new abutment screw conservatively. Rotary instrumentation and/or ultrasonic instrumentation can be used to retrieve the fractured screw carefully. Damaged internal threads of the fixture are avoided in this study. Excessive heat generation at the fixture‐bone interface may cause irreversible bone injury and compromise osseointegration by local necrosis. Delay bone repair affects integration. As a result, clinicians might choose to either remove the fixture or replace it with a new one, or abandon the fixture and cover it with soft tissue.

The expert challenge is required to retrieve this trapped piece. When the methods fail to retrieve the fractured segment, a fixture is considered useless for the patient. This means that another fixture might need to be placed if applicable. This usually involves increased expense, surgical removal of the bone‐integrated dental fixture, and additional procedures, which in turn also modifying the prosthesis design. Different alternatives have been described to deal with a nonrestorable fixture.^21^, ^22^, ^23^ Nevertheless, fixture‐abutment/prosthetic screw retrieval remains challenging and time‐consuming.^24^ In such a scenario, a cast post‐ and core‐supported prosthesis can salvage the near‐useless fixture also.

In this study, we replaced the fabrication of metal post by the screw of the transfer cap of the fixture system itself after adjustment of the height of the piece. This relatively simple technique when executed with the ultimate care and precision would provide excellent result, and the non‐invasive nature of the procedure would also satisfy the patient. Damaged internal threads have been avoided in this procedure.

## 2 MATERIALS AND METHODS

Eight patients were visiting the privet clinic seeking the solution for displaced fixed implant prosthesis. After clinical examination of the site, we discovered that one of the abutment screws in each case was fractured in different levels. Fixtures checked clinically and radiographically, which revealed that all fixtures were osseointegrated very well with a retained fractured screw piece in different grades. All patients have signed an ethical approval for the participation in the research. Table 1 shows descriptive data for each patient.

- Cases with retrieved screw (two patients with an implant‐supported bridge):
  o In the first case, male patient was presented with four fixtures in the upper jaw in the edentulous area from the right lateral to the left second molar bridge (9 units) with single fixture fractured screw that occurs after 7 years postdelivery of the prosthesis.
  o In the second case, female patient was presented with 3 fixtures in the lower mandibular anterior region from left canine to right lateral bridge (5 units) with single fractured screw that occurs after 12 years postdelivery of the prosthesis.

**Table 1:** Clinical description of case series

Pt. No.	Age	Gender	Imp. No.	Bridge unit No.	#screw No.	TT	Period use
1	35	F	4	9	1	Sc. retrieve	7
2	58	F	3	5	2	Sc. retrieve	12
3	55	F	2	3	1	New fix.	5
4	42	M	4	7	1	T. C	4
5	41	F	2	3	1	T. C	8
6	52	M	1	1	1	T. C	0.6
7	26	F	5	10	1	T. C	9
8	72	F	5	7	1	T. C	10

#screw No., number of fractured screw in each patient; Bridge unit No., number of fixed single or multiple bridge units in each patient; Imp. No., number of fixture in each patient; New fix., treatment by new fixture adding; Period use, years that lapsed before fracture screw occurs; Pt. No., number of patient; Sc. retrieve, treatment by retrieving screw; T. C, treatment by replacement of transfer cap screw; TT, treatment option in each patient to solve the problem.

In these two cases, there was previous mobility in the prosthesis occurred first. The screw was retrieved and changed with another one only. A screw was retrieved by an ultrasonic scaler. Care was taken not to touch the threaded walls to prevent any damage for the internal architecture of the fixture. After the fragment was removed, the screw hole was then flushed with dilute aqueous (10:1) sodium hypochlorite to remove any debris and fabrication of a new multiple supported bridges done which occlusally adjusted well.

- A case with a replaced fixture:

One female patient was presented with upper anterior fixed bridge supported by two fixtures in the two central and lateral left side (3 units) after 5 years postfunction. The fractured screw fixture was left, another fixture was placed in the lateral site, and new fixed prosthesis was then fabricated smoothly.

- Cases with a new approach (five patients with a fixture‐supported bridge):
  o In the first case, male patient was presented with 4 fixtures in the upper jaw in the edentulous area from the right canine to the 1st premolar left‐side bridge (7 units) with a single fixture fractured screw that occurs after 4 years postdelivery of the prosthesis.
  o In the second case, female patient was presented with two fixtures in a lower mandibular right‐side region 2nd premolar, 1st, and 2nd molar bridge (3 units) with one fractured screw that occurs after 8 years postdelivery of the prosthesis.
  o In the third case, female patient was presented with single fixture in 1st premolar where fracture screw occurs after six months of use.
  o In the fourth case, male patient was presented with five fixtures in the fully edentulous mandible fixed bridge (10 units) with two fractured screws that occur after 9 years.
  o In the fifth case, older female patient has two fractured screws in the fixed bridge supported by five fixtures in area that extends from left central to right upper first molar fixed bridge (7 units) that occur after 10 years.

A radiograph revealed a well‐integrated fixture with clinically insignificant cervical marginal bone loss by measuring the level of resorption radiographically, with a retained piece of the fractured screws in the opposition that cannot be retrieved. All these cases claimed that there was previous mobility in the prosthesis that occurs first, except one patient has no mobility anteceding the fracture.

Discussions with the patients are maintained to reach the best solution and not to waste the effort to retrieve the fractured piece. The screw hole was flushed with dilute aqueous (10:1) sodium hypochlorite to remove any debris from the fixture.

After discussion of treatment plans with each patient, we assured that it is applicable to use the screw of the transfer cap only to be inserted in the same injured fixture and fabricate the prosthesis. Ethical approval has been signed up by all patients underwent the new policy of treatment.

After agreement with our patients, the following steps are carried out: The screw hole is prepared and flushed with dilute aqueous (10:1) sodium hypochlorite to remove any debris from the fixture; the internal serration of the fixture is checked; the correct diameter of the transfer cap screw check tightness is chosen to fit engagement and then cut the screw to correct height; and then light cure is build up on it. Then, the fabrication of a new prosthesis and postdelivery of the final fixed bridge are performed. Adjustment of the occlusion is done to eliminate any unwanted destructive forces. The patient's occlusal scheme and maximal bite force should be assessed for overload. The new abutment/screw is torqued into the fixture. The crown is occlusally adjusted and recemented for normal functioning. Assessment and correction of the occlusal scheme are important. All patients followed for 6 months to 2 years.

## 3 RESULTS

**Figure 1:**
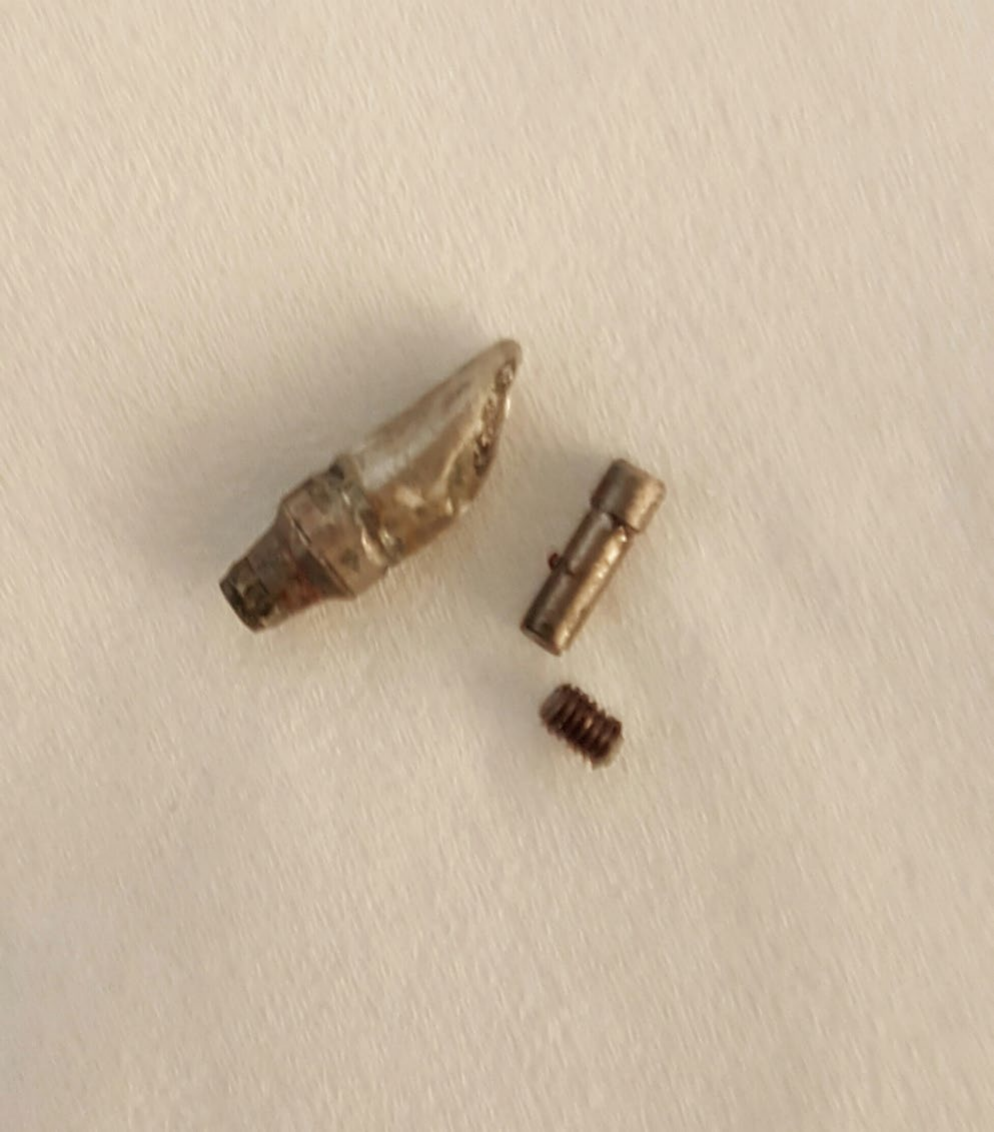
Fractured screw piece removed from patient fixture

**Figure 2:**
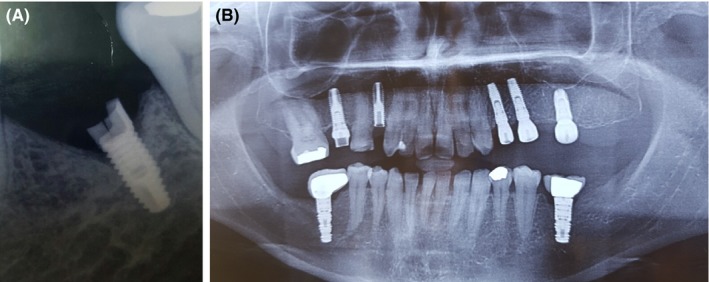
A, Periapical radiograph showing fractured piece stuck in fixture. B, Orthopantomograph showing fractured screw fixture

Abutment screw fractures Figure 1 in bridges supported the fixtures are unhappy events the dentist face in his privet clinic. The patients have a serious complication. It affects esthetic and function as well. Radiographs Figure 2 are important diagnostic aids. According to the level of fracture, the decision whether to proceed a removal of the screw or not is made.

**Figure 3:**
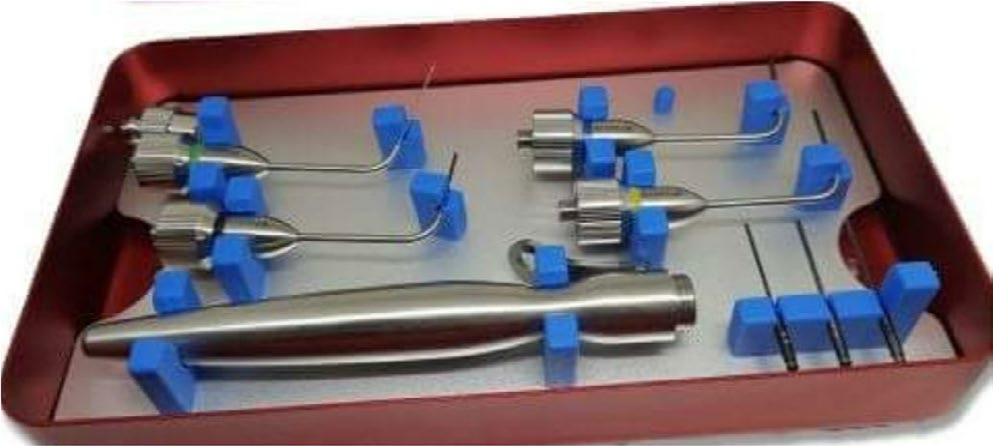
Help Kit used for removal of the fractured screw piece

A cervical part fractured screw is easy to be removed. The removal is carried out either by special kits supported by the Fixtures Company's Suppliers Figure 3 or by using ultrasonic scaler.

**Figure 4:**
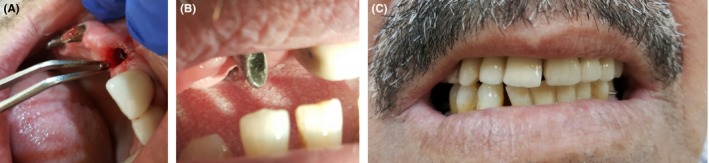
A, Removal of the fractured screw piece from fixture. B, Change of screw only. C, Recementation of fixed prosthesis

The most important need is the skill of the implantologist himself. Removal of fractured piece and change of screw are easily applicable methods in cervical part fractured screw type Figure 4.

**Figure 5:**
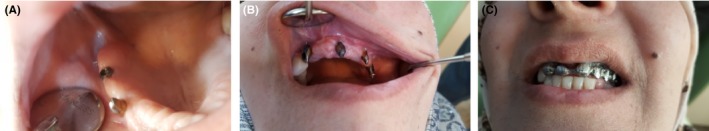
A, Fractured screw. B, Replacement by other fixture. C, Metal frame check for new fixed prosthesis

When all the methods fail to retrieve the fractured segment, the fixture may be rendered useless. So we can solve that problem by placing another fixture according to the normally known protocol Figure 5. Sometimes, this is also difficult for many reasons.

**Figure 6:**
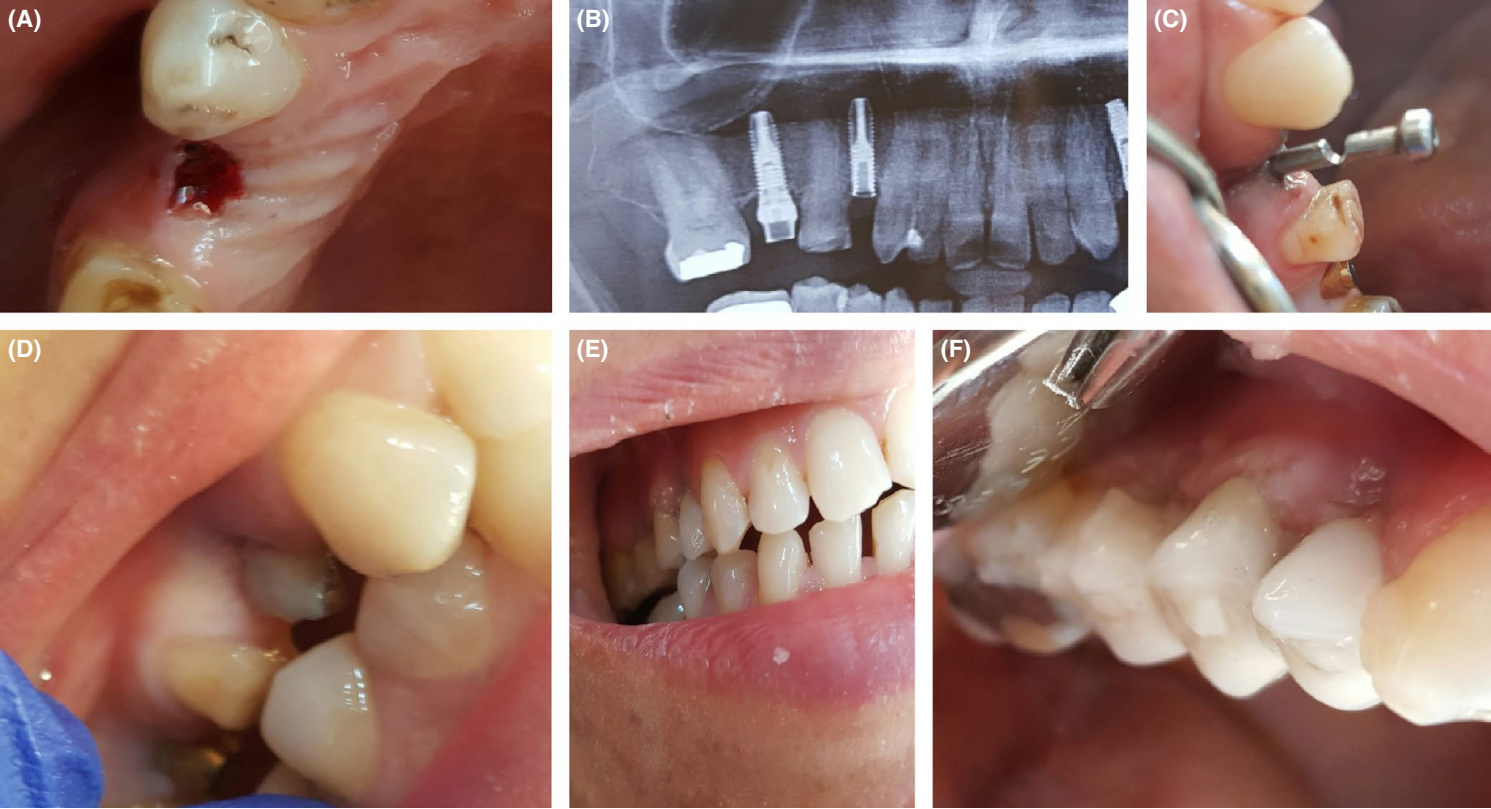
A, Fracture screw site shown clinically. B, Orthopantomograph for fractured screw fixture. C, Transfer cap screw placed in the fixture replacing the abutment screw piece. D, Light cure composite filling that covers the metal transfer cap piece as a core. E, Lateral view of final prosthesis. F, Clear lateral view showing final fixed prosthesis

For such a scenario, we used the transfer cap screw to seat in the space replacing the fractured part instead of the fabrication of metallic custom post and core. By this, we can salvage the near‐useless fixture Figure 6. Therefore, it is a relatively simple technique which would provide an excellent result when executed with ultimate care and precision. Patient follow‐up is mandatory for at least six months to avoid any uncounted complications.

## 4 DISCUSSION

Abutment screw is the part connecting the abutment to the fixture. Fracture screw is one of the beard prosthetic complications in implant dentistry. It is most important as it affects the surgical part which is the fixture, too. We realize that it is rare to occur, but when this occurs actually, challenge is present. Both biomechanics effect and force of mastication should be understood well from the start even before placement of the fixture to avoid such complication.

Apical and middle part fractures need a challenge as it is difficult to be retrieved. Treatments were available to solve this problem different according to many factors. These factors are patient's cooperation and agreement, site available, a cause of fracture, and number of fixture presence at the site. All these factors can change the decision.

Salinas and Oh describe the mechanism in the occlusal loading events applied over the abutment and its retaining screw.^10^, ^11^

There is a slight wobble of the abutment during functional loads. Mechanically fatigue fracture is the main cause of such complication. The crown will generally dislodge, and the apical end of the screw will remain in the fixture.^12^, ^25^

Importantly, after an abutment screw has fractured, assessment of the occlusal scheme is mandatory. Flangan and Lipski advised that the occlusal overloading should be measured in fixture site. Cyclic loading can induce stresses causing an abutment screw fracture, preventing fracture complications can be minimized by occlusal scheme.^26^, ^27^

The fixture screw hole should be flushed with a dilute solution of sodium hypochlorite (0.5%) and rinsed with water to ensure removal of any organic or particulate debris that may inhibit the removal of the fragment.

Radiographical examination added to clinical examination is mandatory in preparation for treatment, to ensure osseous support quality of the supporting fixture and to detect screw‐level fracture. By apical radiographical examination^7^ and by asserting data obtained from clinical examination of site and radiograph, we can approve best treatment option aptly chosen for salvage of the problem.

Seetoh and Yilmaz with their colleagues cite that different brands of fixtures have different abutment screw fractures.^28^, ^29^ Several techniques that are applicable for a fractured abutment screw^19^, ^20^ are as follows:

- Screw fragment retrieval, replaced by another screw.
- Fixture removal or discard and reimplantation in another site if applicable.
- Fabrication of a cemented cast post and core.

Harshakumar et al^13^ dwells that screw loosening and fracture is one of the most common mechanical complications of fixture treatment. We also realized that retrieval of the fractured fragment is challenging when the fracture occurs below the head of fixture or there is damage to its internal threads.

Many (but not all) fixture companies offer fracture screw removal kits (Help Kit). It is expensive and does not consistently remove the fracture segment.^6^ Help Kit Figure 1 includes reverse‐action cutting burs,^30^ but even this can fail to remove the stuck piece. Also, rotary instruments and/or ultrasonic instrumentation^19^, ^20^ can be used to retrieve the fractured screw.

But when all the modalities fail to retrieve the segment, the clinician under such conditions might opt for removing the failed fixture and replacing it with a new one. This gives additional surgical trauma and financial burden to the patient. Thus, salvaging the fixture by other means appears to be a viable option in such situations. Custom cast post and core is presented to solve this complication.

In this study, we assured that it is applicable to use the screw of the transfer cap only to be inserted in the same injured fixture and fabricate the prosthesis. This can be beneficial for the following reasons:

- The injured fixture is not forbidden or discarded.
- There is no need for another surgical intervention to be replaced by a new fixture
- This technique requires less trauma and less cost.
- The fabrication of post and core needs more difficult technical work in both damaging the internal threads of the fixture and fabrication of metal post which is unaccounted future.
- This technique requires less time to fabricate the prosthetic part.

Five patients underwent this procedure from total eight, with good satisfaction of the patients with follow‐up ranged from six months to 2 years. Clinically stationary fixed prosthetic part is well present.

## 5 CONCLUSION

Challenge and luck are always present in such cases. A careful clinical and radiographical examination is mandatory before deciding which line of managing cases with fractured abutment screw is to be used to avoid any uncounted complications. Transfer cap screw can be used gently and cautiously if the fractured piece cannot be retrieved or seated deep in the fixture.

## CONFLICT OF INTEREST

No conflict of interest has been declared as all work has been done in private clinic for all authors.

## AUTHOR CONTRIBUTION

RYA‐R: was a senior specialist in maxillofacial surgery, was the owner of the idea with the official work and patients’ follow‐up, and wrote and published the manuscript. FAM: was a specialist in maxillofacial surgery, drafted and arranged articles, and copied the manuscript. BA‐GT: served as a maxillofacial consultant, critically revised the paper, and contributed to the scientific and professional review.
